# Through your eyes: incongruence of gaze and action increases spontaneous perspective taking

**DOI:** 10.3389/fnhum.2013.00455

**Published:** 2013-08-12

**Authors:** Tiziano Furlanetto, Andrea Cavallo, Valeria Manera, Barbara Tversky, Cristina Becchio

**Affiliations:** ^1^Department of Psychology, Center for Cognitive Science, University of TorinoTorino, Italy; ^2^Department of Psychology, Stanford UniversityStanford, CA, USA

**Keywords:** spontaneous perspective taking, agency, action, gaze, incongruous cues, ambiguous intention

## Abstract

What makes people spontaneously adopt the perspective of others? Previous work suggested that perspective taking can serve understanding the actions of others. Two studies corroborate and extend that interpretation. The first study varied cues to intentionality of eye gaze and action, and found that the more the actor was perceived as potentially interacting with the objects, the stronger the tendency to take his perspective. The second study investigated how manipulations of gaze affect the tendency to adopt the perspective of another reaching for an object. Eliminating gaze cues by blurring the actor's face did not reduce perspective-taking, suggesting that in the absence of gaze information, observers rely entirely on the action. Intriguingly, perspective-taking was higher when gaze and action did not signal the same intention, suggesting that in presence of ambiguous behavioral intention, people are more likely take the other's perspective to try to understand the action.

## Introduction

Near/far, above/below, right/left presuppose a referential center of orientation. Because we cannot separate ourselves from our bodies, it is natural to think that this center of orientation is the body. As Husserl put it, “the ‘far’ is far from me, from my Body; the ‘to the right’ refers to the right side of my Body” (1952/1989). But what happens in presence of others? Are there circumstances where “to the left” with respect to another's body is preferred to “to the right” with respect to my own?

Evidence that the presence of others may change our own coding of spatial locations of objects is provided by recent studies investigating spatial judgment (Tversky and Hard, 2009; Zwickel, 2009; Zwickel and Müller, 2010). In a typical experiment, participants viewed a photograph of two objects on a table. When participants were asked to describe the location of one object relative to another, the dominant response was to adopt their own spatial perspective. If, however, the scene included a person looking or reaching for one of the objects, almost one third of participants spontaneously adopted the other person's perspective, describing the locations from the other's right or left (Tversky and Hard, 2009). These findings indicate that the presence of another person may encourage participants to spontaneously take that person spatial perspective, and describe the locations of the objects from her right or left. Similarly, studies investigating spontaneous visual perspective taking found that observers were slower to make self-perspective judgments when the scene includes a person looking at the scene from a different visual perspective, suggesting that even when the other person's perspective is irrelevant to the task, observers cannot prevent computing the other's perspective (Samson et al., 2010).

What makes people spontaneously take others' perspectives despite the very real presence of their own? The “mere presence” of a human body does not seem sufficient to elicit spontaneous perspective taking (Mazzarella et al., 2012). People adopt the perspective of another person who acts (Frischen et al., 2009; Thirioux et al., 2010) or is positioned to act on objects and even more so when attention is drawn to the person's potential for action, for instance, by phrasing the query about spatial relations in terms of action (e.g., “In relation to the bottle, where does he place the book?” Tversky and Hard, 2009). What is more, people even adopt the perspective of simple geometric shapes when the actions of the shapes appear intentional (Zwickel, 2009).

**Figure 1:**
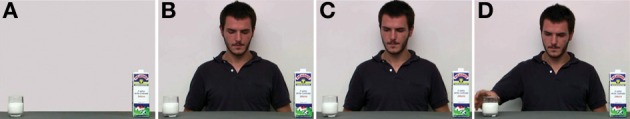
**Final frames for the four videos used in Experiment 1**. In the *No Actor* video **(A)** no actor model was present. In the *Actor* video **(B)** the actor was stationary and looked down, seemingly unaware of the objects on the table. In the *Gaze* video **(C)** the actor turned his head to look toward, but did not reach for the glass. In the *Gaze Action* video **(D)** the actor turned the head to look toward and reached for the glass.

Together, the research suggests that spontaneous perspective taking may be related to understanding and anticipating another's action rather than to the mere presence of a human body. If so, perspective taking should increase when the perceived intention to act increases. This prediction was tested in the first of two experiments. Participants were presented with brief videos (rather than still photographs) depicting two objects, a milk cartoon and a glass full of milk, on a table, with or without a person behind (see Figure 1). Because looking at an object often signals intention to act on the object (Allison et al., 2000; Mennie et al., 2007; Becchio et al., 2008; Pierno et al., 2008; Sartori et al., 2009; Innocenti et al., 2012), the tendency to take the actor's perspective should be stronger when the actor looks at one of the objects and even stronger when the actor reaches toward the object.

Findings concerning the contribution of gaze cues to spontaneous perspective taking have not been consistent. Using static photographs, Tversky and Hard (2009) found no significant difference in the proportion of description from the other's point of view for looking and looking-and-reaching scenes, suggesting that gaze shifts and overt hand actions have similar effects on perspective taking. In contrast, however, Mazzarella et al. (2012) found that the actor's hand action, but not the actor's gaze, modulated the tendency to adopt his perspective. The question remains therefore open as to whether gaze contributes to perspective taking. To address this issue, in a second experiment we manipulated the congruency of gaze and action cues. Gaze cues can be informative but also produce ambiguity with respect to others' actions and behavioral intentions. For instance, football and basketball players often “fake” to fool their opponents, by looking in one direction and acting in another. We predicted that if perspective taking is related to understanding another's action, then, by making the agent's intention ambiguous, incongruous gaze would increase perspective taking.

## Study 1: spontaneous perspective taking increases as perceived intentionality increases

Study 1 was designed to test whether the perceived potential for interaction with objects increases spontaneous perspective taking. We predicted that the more a person is perceived as potentially acting on an object, the greater the need to understand the action, hence in the scene, the stronger the tendency to spatially represent the locations of the objects from the actor's perspective.

### Methods

#### Participants

One hundred and twenty undergraduate students (53 male and 67 female; mean age: 23.5 ± 3.3, range 18–37 years) from the University of Turin volunteered to take part in the experiment. All had normal or corrected-to-normal vision, were right handed, and were naïve with respect to the purpose of the study.

#### Materials and procedures

Participants were presented with one of four videos depicting two objects, a milk cartoon and a glass full of milk, on a table. Scene information was manipulated by introducing an actor model and by varying the actor's gaze and action (see Figure 1). In the *No Actor* video (*n* = 30) no actor model was present. The other three videos included an actor. The *Actor* video (*n* = 30) showed the actor stationary, looking down, seemingly unaware of the objects on the table. In the *Gaze* video (*n* = 30) the actor turned his head to look toward the glass, but did not reach it. In the *Gaze Action* video (*n* = 30) the actor turned the head to look toward and reached for the glass. Videos including the actor started with the actor looking down for 2 s. The actor then turned his head to look at the object (in the *Gaze* and the *Gaze Action* video) and, after 1 s, reached for the object (in the *Gaze Action* video). Each video lasted 4.15 s. The question “In relation to the glass, where is the milk cartoon?” was displayed below the last frame of each video and remained visible until response or until 9 s elapsed. Participants' verbal responses were recorded by the experimenter who was sitting behind the participant.

### Data analysis and results

The responses were scored as 1PP (first person perspective) if the answer was from the participant's point of view, 3PP (third person perspective) if the answer was from the actor's viewpoint, and neutral if the answer gave spatial information from neither perspective (e.g., “next to,” “to the side,” “on the table”). Examples of responses scored as 1PP include: “right,” “on the right,” “to the right from my perspective.” Examples of responses scored as 3PP include: “left,” “to his left,” “to the left from his perspective.” Scored responses were converted into three binary variables for analysis: one variable was coded 1 if the response was 3PP and 0 if it was not; the second variable was coded 1 if the response was 1PP and 0 if it was not; the third variable was coded 1 if the response was neutral and 0 if it was not. To assess the influence of agency cues on spontaneous perspective taking, separate binary logistic regression analyses were conducted on 3PP, 1PP, and neutral responses. The type of video (*No Actor, Actor, Gaze, Gaze Action*) was entered as independent variable of interest.

**Figure 2:**
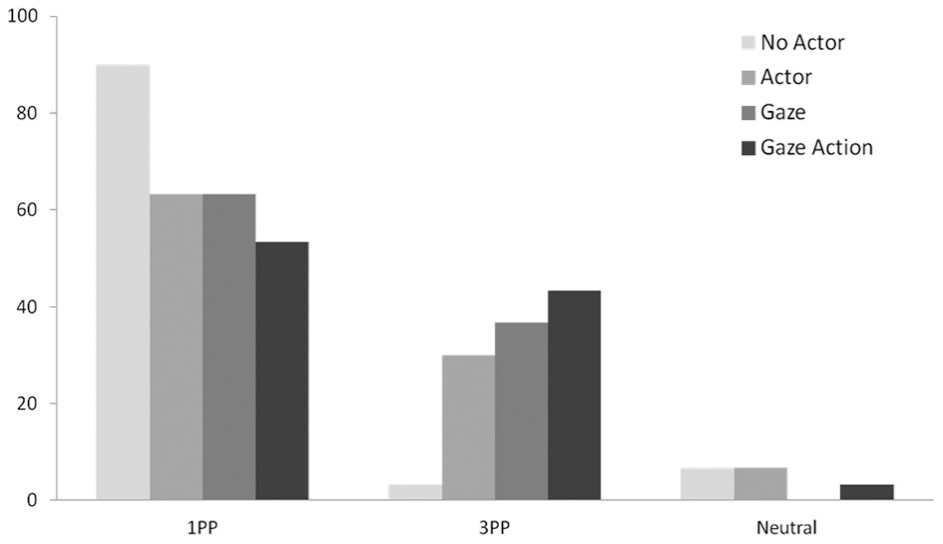
**Percentages of 1PP, 3PP, and neutral responses in Experiment 1**.

In line with predictions, binary logistic regression analysis on 3PP responses yielded a significant linear effect of agency (Wald χ^2^ = 10.903, *df* = 1, odd ratio = 1.968, CI = 1.317–2.941, *p* = 0.001). The percentage of 3PP responses was highest for *Gaze Action* video (43.3%), lower for the *Gaze* video (36.7%), and even lower for the *Actor* video (30%, see Figure 2). For the *No actor* video, only one participant adopted the 3PP perspective. Similarly, the percentage of 1PP responses was affected by agency cues (Wald χ^2^ = 7.872, *df* = 1, odd ratio = 0.591, CI = 0.409–0.853, *p* = 0.005). The percentage of 1PP responses was highest for the *No Actor* video (90%), lower for the *Actor* video (63.3%) and the *Gaze* (63.3%) video, and lowest for the *Gaze Action* video (53.3%, see Figure 2). The percentage of neutral responses was not affected by agency cues (Wald χ^2^ = 0.993, odd ratio = 0.645, CI = 0.272–1.528, *p* = 0.319).

Together, these findings corroborate and extend the idea that increased potential for interaction enhances spontaneous perspective taking.

## Study 2: spontaneous perspective taking increases as incongruity of intention increases

Gaze is an important source of information about others' intentions and actions (Allison et al., 2000; Mennie et al., 2007; Becchio et al., 2008; Pierno et al., 2008; Sartori et al., 2009; Innocenti et al., 2012). From the gaze of another person, we can infer what the person is interested in, what she might desire, and, consequently, what she will do next (Pierno et al., 2006). Gaze direction, however, can also produce ambiguity with respect to the other's intention. This can occur, when gaze conveys conflicting information with respect to the behavioral intention of the agent (Hudson and Jellema, 2011). In this situation, the agent's action can be perceived as ambiguous and observers might be encouraged to adopt the perspective of the other person to understand her intention. Spontaneous perspective taking might thus be expected to be even stronger when gaze is incongruous than when gaze and action signal the same, and therefore unambiguous, intention.

To test this prediction, in Study 2, we presented participants with videos of an actor reaching for a glass in presence of a milk cartoon. The actor either looked toward the glass before reaching (*Gaze Action*) or reached without looking (*Ambiguous Gaze Action*). We predicted that the absence of a shift of gaze in the direction of action would make the action harder to understand and therefore increase the likelihood of adopting the actor perspective. In contrast, no increase in perspective taking should be expected when access to the actor's gaze during reaching is prevented by blurring the actor's face (*Blurred Gaze Action*). This is because, in this situation, the absence of gaze cues does not render the agent's behavioral intention ambiguous.

### Methods

#### Participants

Based on the prevalence of 3PP/1PP responses for the *Gaze Action* scene compared to the *Actor* scene and the *Gaze* scene in Experiment 1 (9.7%), we estimated that we would need 135 participants in each condition to evaluate the effect of gaze manipulations on 3PP and 1PP responses (see Supplementary Material). Four hundred and five undergraduate students (191 male and 214 female; mean age: 23.3 ± 3.3; range 18–48 years) from the University of Turin were thus recruited to take part in Experiment 2. All had normal or corrected-to-normal vision, were right handed, and were naïve with respect to the purpose of the study.

#### Materials and procedures

**Figure 3:**
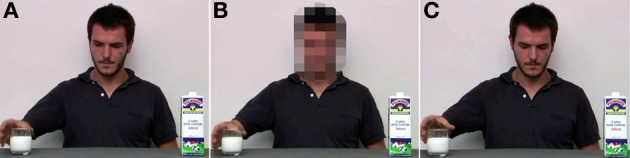
**Final frames for videos in Experiment 2**. In the *Gaze Action* video **(A)** the actor turned the head to look toward and reached for the glass. In the *Blurred Gaze Action* video **(B)** the actor turned his head, looked toward and reached for the glass but participant's access to the actor's gaze direction was prevented by blurring the actor's face. In the *Ambiguous Gaze Action* video **(C)**, the actor reached the glass without looking toward it.

Procedures were the same as those in Study 1, except that participants were presented with one of three videos depicting an actor reaching for one of two objects—a milk cartoon and a glass full of milk—on a table (see Figure 3). In the *Gaze Action* video (*n* = 135) the actor turned his head, looked toward and reached for the glass (see Study 1). In the *Blurred Gaze Action* video (*n* = 135) the actor turned his head, looked toward and reached for the glass as in the *Gaze Action* video. Access to the actor's gaze direction was, however, prevented by blurring the actor's face. In the *Ambiguous Gaze Action* video (*n* = 135) the actor reached for the glass without looking at it.

### Data analysis and results

As in Study 1, the responses were scored as 1PP if the answer was from the participant's point of view, 3PP if the answer was from the actor's viewpoint, and neutral if the answer gave spatial information from neither perspective. Separate chi square analyses were conducted to compare observed frequencies of 3PP (vs. 1PP and neutral responses), 1PP (vs. 3PP and neutral responses), and neutral responses (vs. 1PP and 3PP responses) for the *Ambiguous Gaze Action* and for the *Blurred Gaze Action* scenes with expected frequencies for the *Gaze Action* scene.

**Figure 4:**
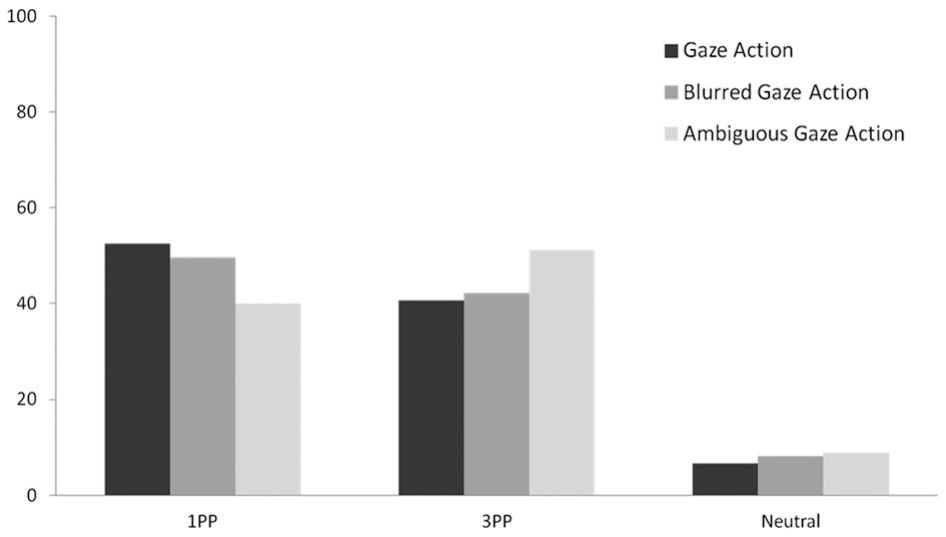
**Percentages of 1PP, 3PP and neutral responses in Experiment 2**.

Strikingly, when the actor reached without looking, 51.1% of the participants adopted his perspective (see Figure 4). Chi-square analysis revealed a marginally significant increase in 3PP responses for the *Ambiguous Gaze Action* scene compared to the *Gaze Action* scene (51.1% vs. 40.7%; χ^2^ = 3.713, *df* = 1, *p* = 0.054, *r* = 0.166). Conversely, 1PP responses were significantly lower for videos in which the actor reached without looking than for videos in which reaching was preceded by looking (40% vs. 52,6%; χ^2^ = 8.586, *df* = 1, *p* = 0.003, *r* = 0.252). Taken together, these findings suggest that perspective taking was increased for the *Ambiguous Gaze Action* scene compared to the *Gaze Action* scene. As predicted, *Blurred Gaze Action* and *Gaze Action* videos yielded equivalent percentages of 3PP (42.2% vs. 40.7%; χ^2^ = 0.112, *df* = 1, *p* = 0.738, *r* = 0.029) and 1PP responses (52.5% vs. 49.6%; χ^2^ = 2.478, *df* = 1, *p* = 0.115, *r* = 0.029). Neutral responses were neither affected by the ambiguity of actor's intentions (6.6% vs. 8.8%; χ^2^ = 1.071, *df* = 1, *p* = 0.301, *r* = 0.089) nor by the gaze blurring (6.6% vs. 8.1%; χ^2^ = 0.476, *df* = 1, *p* = 0.490, *r* = 0.059).

## General discussion

Converging evidence from social neuroscience suggests that people use knowledge of their own bodies to understand other people's behavior (Grafton, 2009). Accordingly, understanding of others' actions, intentions, and emotions has been proposed to rely on mechanism of embodied simulation (e.g., Becchio et al., 2012). Together with previous research (e.g., Tversky and Hard, 2009), the present results suggest that, in the service of action understanding, people may also embody others' location, spatially representing the world from others' point of view rather than from their own.

### Agency cues in video displays

The mere presence of another person in a position to act on objects encouraged about 30% of respondents to take the other person's perspective. Critically, as demonstrated in Study 1, the tendency to take the actor's perspective increased when the actor looked at one of the objects (36.7%) and became even stronger when the actor reached for one object (43.3%). This corroborates the interpretation that perspective taking increases to the extent that the person is perceived to be potentially interacting with the objects.

Previous studies investigating spontaneous perspective taking have reported considerably lower percentages of third-person responses for looking and reaching scenes then those reported here (e.g., 22 and 29%, respectively; Tversky and Hard, 2009). One aspect of the present study that is likely to have contributed to increase perspective taking is the use of videos instead of photographs. Videos provide dynamic cues to action not available in static displays. As human observers are particularly sensitive to human body movements (Blake and Shiffrar, 2007), it is plausible that the gradual unfolding of action emphasizes and draws attention to action, thereby increasing perspective taking.

A question for future research is whether perspective taking is further encouraged by the observation of actions potentially directed at the observer. Social cognition has been proposed to be substantially different when we are in interaction with others (second-person interaction) rather than merely observing them (third-person interaction; Schilbach et al., 2013). Second-person interaction modulates emphatic brain responses (Singer et al., 2006) and there is evidence that simulation of another person's action, as reflected in the activation of the observer motor system, gets stronger the more the other is perceived as an interaction partner (Kourtis et al., 2010). In terms of perspective taking, observation of the actions of a potentially interacting partner might thus be expected to elicit stronger perspective compared to observation of the actions of a third party we do not interact with.

### When looking is ambiguous

Mazzarella et al. (2012) reported that action triggered perspective-taking, but gaze cues did not. They suggests that this may be because eye gaze is not critically relevant, as grasping is, to understanding what an actor is currently doing. However, other research has shown that other gaze direction is informative not only about *future intentions* but also about *present intentions* and *motor intentions* and can change the way current actions are perceived (Pierno et al., 2008). Reaching is typically guided by the eyes. Gaze leads the hand to the object to be grasped and supports predictive motor control in manipulation (Johansson et al., 2001). Observing a person grasping without looking may thus be perceived as ambiguous. What is he planning to do? Why is he not looking at the object he is reaching for? In Experiment 2 we found that compared to a situation in which gaze and action signaled the same intention, perspective taking increased for reaching without looking, apparently in an effort to understand the intended action in the face of conflicting cues. In contrast, we observed no increase in perspective taking when looking cues were eliminated by blurring the eyes, suggesting that when there was no conflict, observers used the direction of reaching as a cue to understand the intention.

Allocation of attention to gaze cues is a flexible process that depends in part on the perceived ambiguity of an agent's intentions. Observers do not attend to an agent's gaze direction automatically, but rather do so when other social cues are insufficient to determine the immediate course or goal of the action (Hudson and Jellema, 2011). The present findings suggest that similarly to attention, perspective taking may not be triggered directly by the perceptual properties of gaze stimuli, but may depend on gaze intentional significance in the overall context. When gaze and action cues convey the same information, gaze processing adds little to action in terms of intention attribution. Eliminating gaze cues has thus no influence on perspective taking. However, when gaze and action convey incongruous information making the agent's intention ambiguous, gaze direction becomes relevant and may increase spontaneous perspective taking. These findings may help to reconcile inconsistent findings concerning the relative contributions of gaze and action cues to perspective taking (e.g., Tversky and Hard, 2009; Mazzarella et al., 2012) by showing that, rather than depending on specific bodily cues (and not others), perspective taking is influenced by the attribution of intentions to others.

## Conclusions

Here, participants watched videos of two objects on a table under varying conditions. They were asked to report the spatial relations between the two objects. When only the objects were in the scene, participants responded from their own viewpoint. However, when the scene included an actor in the position to act on the objects, participants frequently took the actor's perspective. The first study showed that the more the actor was perceived as potentially interacting with the objects, the stronger the tendency to take his perspective. The second study investigated how manipulations of gaze affect the tendency to adopt the perspective of another reaching for an object and found that perspective-taking increased when gaze and reaching information was incongruous making the agent's behavioral intention ambiguous. These findings add further support to the idea that spontaneous perspective taking is in the service of action understanding. When the action is more difficult to understand, there is more perspective taking. It is as if observers are putting themselves in the place of the actor to understand what he is intending to do.

But why would someone spontaneously take the spatial perspective of another when the other appears to be engaged in action? Interacting with others, understanding what they are doing and what they are likely to do next all require some comprehension of what the world looks like to them. As suggested previously (Tversky and Hard, 2009), taking the perspective of the other may be effective for planning a response to others' actions, but also for learning by observation. What makes the current results surprising is that the action was mundane—so no need to learn by observation—and required no complementary action in response. Even more surprising is that the perspective was expressed in language, in the especially confusable terms, “left” and “right,” which are well-known to take more time to produce and to produce more errors than other directional terms like “front” and “back.” Despite this, when the agent's intention was ambiguous, the majority of participants spontaneously adopted the agent's perspective rather than their own.

### Conflict of interest statement

The authors declare that the research was conducted in the absence of any commercial or financial relationships that could be construed as a potential conflict of interest.
