# Supplementary material for: Through your eyes: incongruence of gaze and action increases spontaneous perspective taking
Source: Front Hum Neurosci. 2013 Aug 12;7:455. doi: 10.3389/fnhum.2013.00455 (PMC3740297; doi:10.3389/fnhum.2013.00455)
Supplement: Supplementary file 1 [file 56365__Data_Sheet_1.DOCX]

**Experiment 2**

**SUPPLEMENTARY DATA ANALYSIS AND RESULTS**

**Sample size estimation**

To estimate the number of participants that we would need in each condition to evaluate the effect of gaze manipulations on 3PP and 1PP responses, we employed the following equation:

$$n=\frac{t^{2} P (1-P)}{D^{2}}$$

where t is the value from the standard normal distribution reflecting the confidence level that will be used, P the estimate of prevalence of 3PP/1PP responses, and D the desired level of absolute precision.

In Experiment 1 the mean increase in 3PP responses for the *Gaze Action* scene compared to the *Gaze* scene and the *Actor* scene was 9,5%. Similarly, the mean decrease in 1PP responses for the *Gaze Action* scene compared to the *Gaze* scene and the *Actor* scene was 10%.

Based on these data, we used P=0.097 and D^2^=0.05 to estimate the sample size and obtained n=135.

**Percentages of 1PP, 3PP and neutral responses for** **subgroups**

Chi-square analysis revealed a marginally significant increase in 3PP responses for the Ambiguous Gaze Action scene compared to the Gaze Action scene (51,1% vs. 40,7%; χ² = 3.713, df = 1, p = .054). Conversely, 1PP responses were significantly lower for videos in which the actor reached without looking than for videos in which reaching was preceded by looking (40% vs. 52,6%; χ² = 8.586, df = 1, p = .003). In line with predictions, the increase in 3PP responses (and the corresponding decrease in 1PP responses) is thus around 10%. A similar increase/decrease is observed when considering the first 30 participants in each condition and when dividing participants in 4 subgroups (30 + 30 + 45) in each condition:

|  | Participants | | | |
| --- | --- | --- | --- | --- |
| 3PP responses | 001-030 | 031-060 | 061-090 | 091-135 |
| **Gaze action** | 43,33% | 43,33% | 40% | 39,77% |
| **Ambiguous Gaze Action** | 56,66% | 50% | 46% | 51,11% |
|  |  |  |  |  |
| 1PP responses | 01-030 | 031-060 | 061-090 | 091-135 |
| **Gaze action** | 53,33% | 50% | 46,66% | 57,77% |
| **Ambiguous Gaze Action** | 40% | 40% | 40% | 40% |

This indicates that the Ambiguous Gaze Action manipulation produced a rather small but consistent effect on perspective taking.
